# The potential effects of cranberry extract on indomethacin-induced gastric ulcer in rats

**DOI:** 10.12688/f1000research.158944.2

**Published:** 2025-06-10

**Authors:** Zaid Abdul-Majeed, Mohammed Qasim Yahya Malallah A. Al-Atrakji

**Affiliations:** 1 Department of Pharmacology, College of Medicine, University of Baghdad, Baghdad, Baghdad Governorate, +964, Iraq

**Keywords:** Cranberry extract; Omeprazole; Gastric ulcer; Indomethacin

## Abstract

**Background:**

Indomethacin belongs to nonsteroidal anti-inflammatory drugs (NSAIDs) prescribed for treatment of rheumatoid diseases and linked to the development of gastric ulcers in many people. Cranberry is a rich source of polyphenols and flavonoids, which have powerful antioxidant and anti-inflammatory properties.

**Methods:**

This study aimed to evaluate the activity of cranberry aqueous extract on indomethacin-induced gastric ulcers in albino rats. 20 adult male rats were sequentially assigned to four groups of 5 each. The control group consumes distilled water (DW) orally for 15 days. The induction group received a single oral dosage (60 mg/kg) of IND. The omeprazole group got 60 mg/kg of indomethacin as a single oral dose and then 20 mg/kg/day of omeprazole for 15 days. The cranberry group was given a single dose of indomethacin 60 mg/kg orally and subsequently 200 mg/kg/day of cranberry aqueous extract for 15 days. Rats were euthanized on day 15, and gastric tissues were removed for biochemical and histopathological evaluations.

**Results:**

Cranberry extract considerably ameliorated the severity of indomethacin-induced gastric ulcerations and fixed histological deteriorations. Furthermore, indomethacin-exposed rats treated with cranberry extract exhibited dramatically lower serum levels of inflammatory biomarkers like TNF-α and IL-6, but higher levels of anti-oxidative biomarkers like SOD and GPx. The bioactive flavonoids and polyphenols content of cranberry extract could possibly account for its profound gastroprotective effects. The anti-oxidative and anti-inflammatory properties of cranberry extract could be a promising strategy for ameliorating the indomethacin-aggravated gastrotoxicity.

## Introduction

Despite recent advancements in pharmaceutical technology, the gastrotoxicity of non-steroidal anti-inflammatory drugs (NSAIDs), which frequently result in stomach ulcers and delayed healing, continues to be a significant issue. ^1, 2^ Gastric ulcers caused by the use of NSAIDs are caused by several factors, including the inhibition of prostaglandin-E2 (PGE2) or angiogenesis, the enhancement of the generation of free radicals, the induction of cyclooxygenase-2 (COX-2) expression, and the production of cytokines that are responsible for pro-inflammatory effects. ^3– 5^ In addition, it has been demonstrated that NSAIDs decrease the rate at which ulcers heal by inhibiting the development of pro-angiogenic factors. ^6^ These pro-angiogenic factors include vascular endothelial growth factor (VEGF), platelet-derived growth factor (PDGF), and essential fibroblast growth factor (bFGF). ^7– 12^ Indomethacin (IND) was found to have a more significant propensity to induce damage to the stomach than traditional NSAIDs. ^13^

Omeprazole, which is regarded as an important drug by the World Health Organization (WHO), is utilized extensively throughout the world to treat a variety of gastrointestinal conditions. Omeprazole inhibits the production of stomach acid by inhibiting a proton pump inside the stomach. ^14^ Furthermore, it lowers the levels of endogenous oxidative stress as well as cytokines that promote inflammation. ^15^ In recent years, several natural products have been introduced that, due to their anti-oxidative qualities, make it possible to ease gastrointestinal illnesses. ^16^ In this regards, several illnesses were effectively managed with botanical medicine, which entails the administration of plant-derived products. ^17– 20^ The natural therapies are safer and display minimal undesirable effects than manufactured drugs. ^21– 25^

Natural substances and their medically active elements are extensively researched and evaluated as possibly successful therapies for a variety of diseases. ^26– 31^ These substances are being used to offer gastro-protection in a variety of contexts, notably the avoidance of chronic progressive gastric disease and acute stomach ulcers. ^32^

Cranberries, also known as Vaccinium macrocarpon, are an important source of phytochemicals, particularly polyphenols, citric and malic acids, vitamin C, and triterpenoids, which are required for the antioxidant activity of the fruit. ^4, 33^ In addition to phenolic acids and benzoates, the red cranberry contains a high concentration of flavonoids, specifically proanthocyanins, anthocyanidins, and flavanols. ^34, 35^ There are also phenolic acids and benzoates present. Cranberry can treat a variety of other problems, including gingivitis, diarrhoea, and cardiovascular health. ^36^ To the best of our knowledge, no previous studies were carried out to investigate the impact of cranberry extract on NSAID-induced gastric ulceration. This study aimed to evaluate the efficacy of cranberry extract in treating indomethacin-induced gastric ulcers in albino rats by exploring its effects on antioxidant enzymes, inflammatory markers, and histological findings. Furthermore, the possible mechanisms underlying its therapeutic effects are being investigated.

## Methods

### Materials

**Experimental animals**

Twenty male albino rats aged between 6 and 12 months with an average weight of 200 ± 15 g, were used in this study. The animals were acquired from the animal house of the Iraqi Centre for Cancer Research and Medical Genetics– Baghdad – Iraq and housed in it. They were placed in polyethylene cages with stainless steel covers and raised to prevent coprophagy. Rats were kept for acclimatization for one week before the experiment. They were maintained in standard laboratory conditions (25°C, 12-hour light-dark cycle) and had free access to food from a chow pallet and tap water. They fasted for 24 hours before indomethacin admiration and were allowed free access to water. The study was started at the beginning of January 2024 and finished in February 2024. This study was approved by the ethical committee for experimental studies at the College of Medicine/University of Baghdad.

**Drugs and reagents**

Indomethacin 60 mg/kg was purchased from Sigma Aldrich; Omeprazole 20/kg mg was obtained from its capsules marked by Acino, Zurich, Switzerland. Cranberry Extract was obtained from its tablet, Adrien Gagnon, Canada, and sodium carboxymethylcellulose was obtained from Loba Chemie, India.

## Methods

### Preparations of pharmaceutical solutions

According to previous studies, an oral administration of 60 mg/kg of indomethacin is needed to induce gastric ulcers. ^3, 4^ The suspension had a concentration of 24 mg/ml. Experimentation requires a volume of 0.5 mL to provide a dose of 60 mg/kg to rats.

Regarding omeprazole, previous studies relied on an oral dose of 20 mg/kg. ^37^ An oral suspension was prepared for administering omeprazole orally. The suspension was made with 0.5% Na-CMC as the suspending agent, and omeprazole capsules (20 mg) were used as the source of the active component. The suspension had a concentration of 8 mg/ml. To provide a dose of 20 mg/kg for experimental rats with an average weight of 200 ± 15 g, a volume of 0.5 ml is required.

To administer cranberry aqueous extract orally, an oral suspension was made by utilizing 0.5% Sodium salt of carboxymethylcellulose (Na-CMC) as a suspending agent and cranberry extract tablets (270 mg) as a source of the active ingredient. The amount of cranberry extract was calculated based on the results of an oral acute toxicity study, ^38^ in which rats were given a dose of 2000 mg/kg. It showed no evidence of toxicity in their bodies. Therefore, the study utilized one-tenth of this dose, which is equivalent to 200 mg/kg, to validate the safety of the substance, as stated by another study. ^39^ An oral suspension of the powder was made by combining powder cranberry extract with 0.5% Na-CMC to provide a concentration of 80 mg/mL. For experimental rats with an average weight of 200 ± 15 g, a volume of 0.5 ml is necessary to provide a dose of 200 mg/kg according to standard protocols.

### Experimental design

This research project was carried out in the Department of Pharmacology, College of Medicine, University of Baghdad, as well as the Iraqi Laboratory for Cancer and Biomedical Genomic Research. The current examination began on January 10, 2024 and lasted around June 30, 2024. Experimental rats were randomly assigned into four groups with each group consisting of five animals as the following:

• Group 1(n=5) is the standard control group, which was kept under normal laboratory conditions and received 0.5 mL of oral 0.5% Na-CMC suspension for 15 days by oral gavage.
• Group 2(n=5) is the ulcer induction group, which received 60 mg/kg of indomethacin at day 0 and oral 0.5% Na-CMC suspension for 15 days by oral gavage.
• Group 3(n=5) is the standard oral Omeprazole-treated group, which received 60 mg/kg of indomethacin at day 0 and omeprazole oral suspension (20 mg/kg) in 0.5% Na-CMC for 15 days by oral gavage.
• Group 4(n=5) is the cranberry extract-treated group, which received 60 mg/kg of indomethacin at day 0 and cranberry extract oral suspension (200 mg/kg) in 0.5% Na-CMC for 15 days by an oral gavage.

### Tissue and blood sample collection

The animals were put under anesthesia with 87 mg of ketamine/kg of body weight and 13 mg of xylazine per kg at the end of the experiment, which was day 15. ^40– 42^ Every attempt was established to reduce the overall number of animals employed for the experiments and minimize their misery by keeping them in private, clean boxes with a broadened metal mesh floor beneath appropriate spots, making sure they had a 12-hour span of daylight and darkness, and giving them anesthetic medications to ease any kind of discomfort or pain they may have experienced. The samples of blood were taken by performing a direct heart puncture with plastic syringes containing 5 milliliters, and then they were placed into gel tubes. The tubes were then subjected to centrifugation for ten minutes at a speed of three thousand revolutions per minute throughout the entire process. ^43– 47^ After the complete separation of the blood, the serum was removed, then deposited into plastic tubes with a capacity of 2 mL that had not been treated, and then stored at a temperature of -20 °C for further analysis. ^48– 51^

### Assessment of inflammatory and anti-oxidative parameters

The serum concentrations of superoxide dismutase (SOD) and glutathione peroxidase (GPx), as well as tumor necrosis factor-alpha (TNF-α) and interleukin-6 (IL-6), were measured for all experimental groups ^1– 4^ after 15 days of the experiment. The ELISA kits employed in this investigation were generally obtainable and were prepared according to the supplier’s directions (Elabscience ^®^ Laboratory, China). The first part of the procedure is to add anti-marker antibodies to a plate with 96 holes. The tubes were filled with specimens and standards, and the packaging antibodies drew any TNF-α, IL-β, SOD, or GPx from the circulation. Following detaching the unpaired biotin-associated antibody, streptavidin and horseradish peroxidase (HRP) were properly applied to the prepared plates. ^52– 57^ The total amount of indicators in each collection was estimated by comparing optical density to traditional charts. The plates were cleaned once again, and TMB-substrate blends were used to show the matched marker quantities utilizing the resulting color. The absorbance of the specimens was determined employing a microplate reader spectrophotometer. The color intensity is calculated at 450 nm when the color changes from blue to yellow with a stop solution. ^58– 63^

### Haematoxylin and eosin staining

Gastric tissue slices from different rat groups have been treated with hematoxylin and eosin. The technique involves warming aluminum potassium sulfate and dispersing it in purified water. Hematoxylin was submerged in alcohol concurrently. Following heating, the two mixtures were incorporated and withdrawn from heat. A tiny quantity of mercuric oxide was progressively poured while spinning before being immersed in frigid water. ^64– 68^

2 grams of eosin powdered form were dissolved in 25 millilitres of distilled water, and then 475 millilitres of 100% alcohol were added to produce 0.5 litters of eosin. It produces reddish or pink colors in the cytoplasm and components of the extracellular matrix. ^69– 72^

### Assessment of histopathological changes

The stomachs of all experimental groups ^1– 4^ were taken on the 15th day of the trial. Following the administration of ketamine and xylazine anaesthesia, the stomachs of the animals were extracted and preserved in 10% formalin for histological inspection. ^73– 76^ Afterward, the samples were immersed in paraffin, cut into slices that were 5 μm in thickness, and then treated with haematoxylin and eosin (H&E) stain. ^77– 80^

The histological slides were analyzed using standard light microscopy techniques. An experienced pathologist examined the treatment group without any prior knowledge, and only one sample slide was selected for each group.

The semi-quantitative score based on inflammation severity (0-3), haemorrhagic spot (0-3), sub-mucosal edema (0-3), and superficial mucosal ulcers (0-3) was used to assess changes in the stomach tissue between the experimental groups after 15 days for comparison purposes. ^81– 84^

### Statistical analysis

Statistics were done in Graph Pad Prism 9. Data were introduced in SPSS version 22 (Statistical Program for the Social Sciences). Mean SD is employed to determine descriptive statistics. A statistical assessment was conducted, and various charts and tables were prepared to account for unanticipated variables. To determine group associations, ANOVA and post hoc Tukey’s multiple-comparisons tests were used. Statistical significance required a P-value below 0.05. The histopathological scoring system employed median and interquartile ranges, but all other data were presented as mean and standard deviation. To analyze histological group scores, Dunn’s multiple comparisons test followed the non-parametric Kruskal-Wallis test. ^85^

## Results

**Figure 1.**
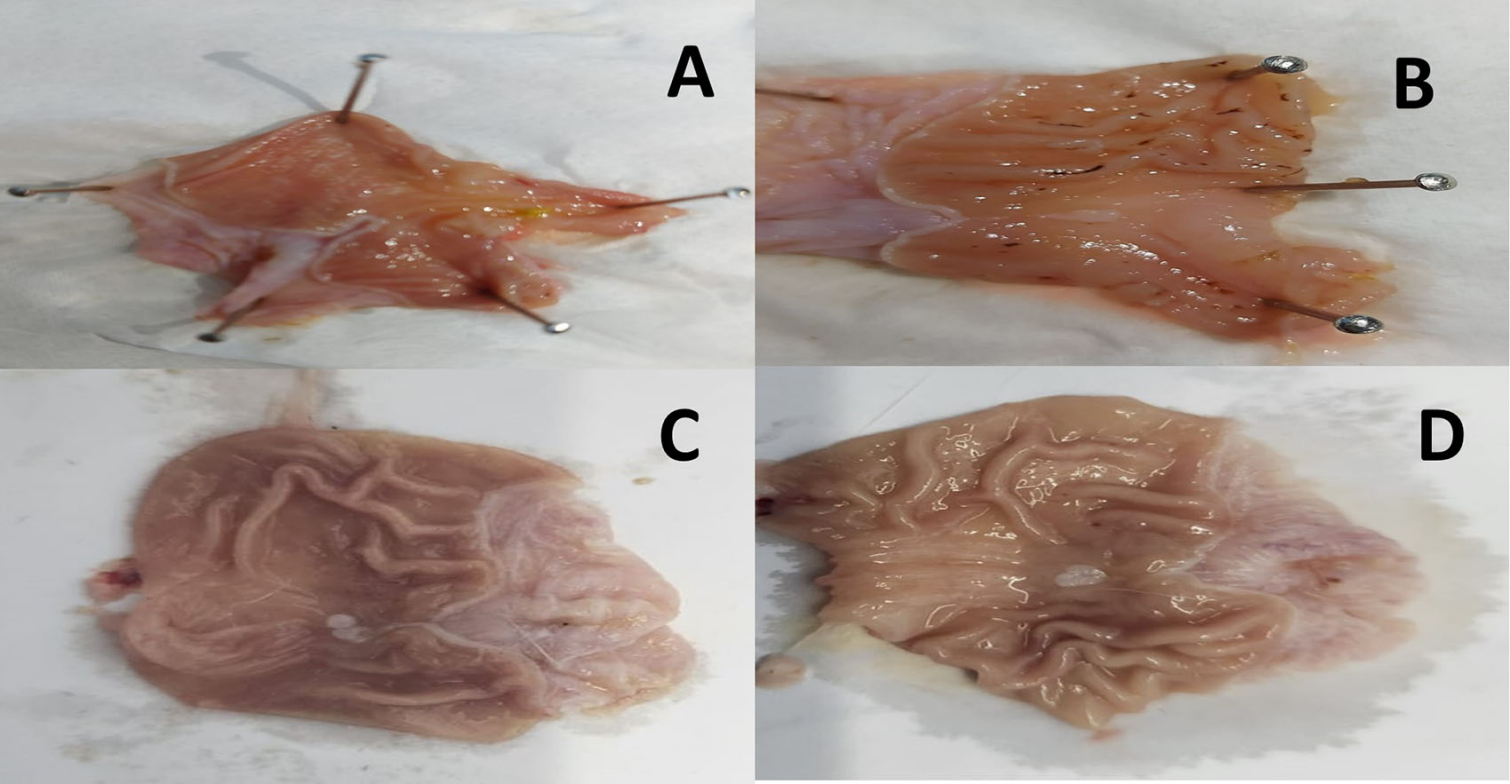
Representative photographs of the macroscopic structure of the stomach in rats. A = Normal control group (G1), B = Indomethacin group (G2), C = Omeprazole group (G3), D = Cranberry treatment group (G4).

The results revealed an apparent induction of peptic ulcer with considerable macroscopic alterations in gastric tissue sections of the indomethacin group (G2) compared to the control group (G1). However, both the omeprazole (G3) and cranberry extract (G4) groups exhibited fewer macroscopic stomach modifications than the indomethacin group, culminating in a much lower severity and extent of the experimentally generated ulcer, as depicted in Figure 1.

**Figure 2.**
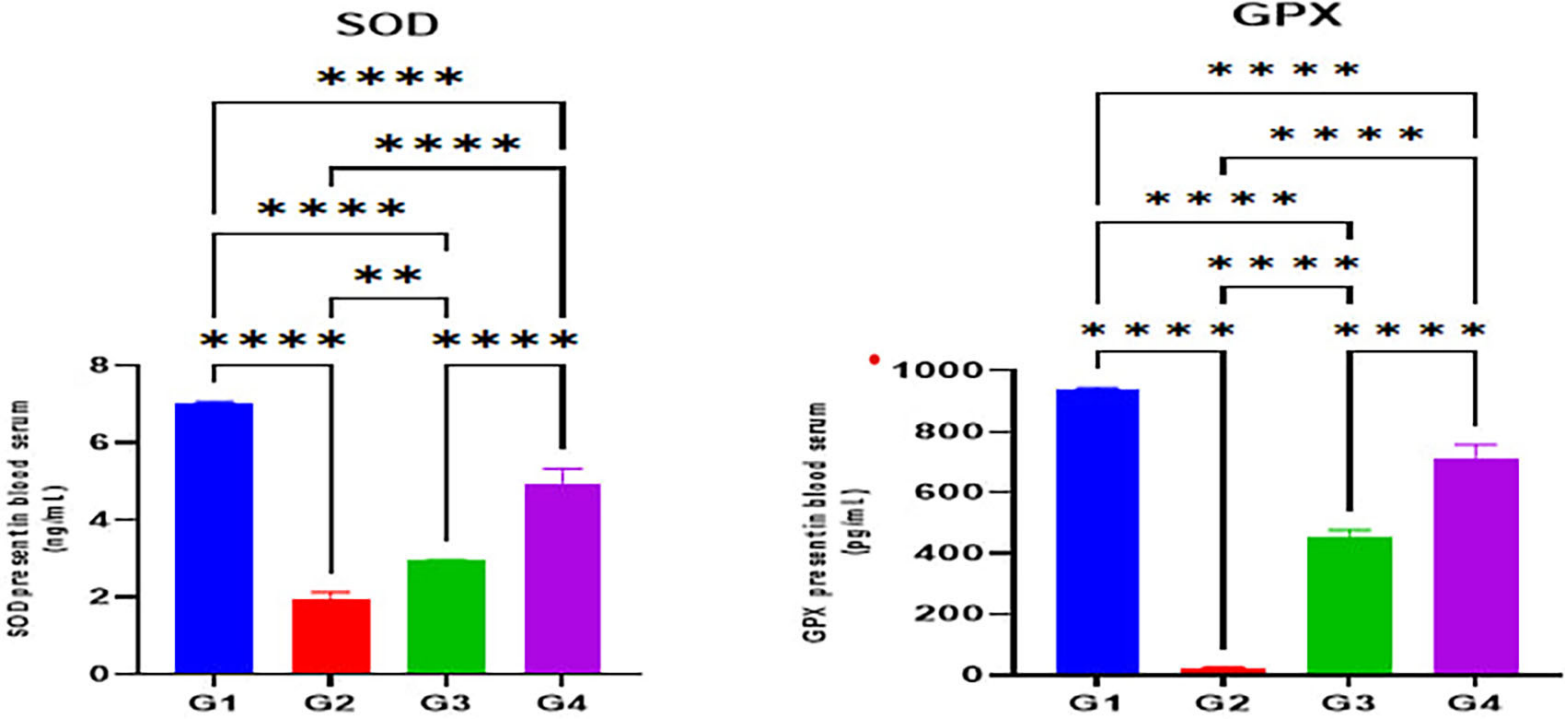
Effects of studied drugs on serum levels of antioxidant enzymes (SOD and GPX) estimated at day 15 of the experiment for the control (G1), indomethacin induction (G2), omeprazole treatment (G3), and cranberry treatment (G4) groups. Data were presented as Mean±SD; ****= significant differences (p < 0.05), n = 5 animals/group.

The indomethacin group (G2) disclosed significantly reduced serum levels of antioxidant enzymes SOD and GPX in comparison with the normal control group (G1) (p < 0.05). Nonetheless, omeprazole (G3) and cranberry (G4) treatment groups demonstrated significantly higher serum levels of antioxidant enzymes SOD and GPX in comparison with the indomethacin induction (G2) group (p < 0.05). However, there were no substantial differences in SOD or GPX levels between the omeprazole (G3) and cranberry extract (G4) groups (P > 0.05) as seen in Figure 2.

**Figure 3.**
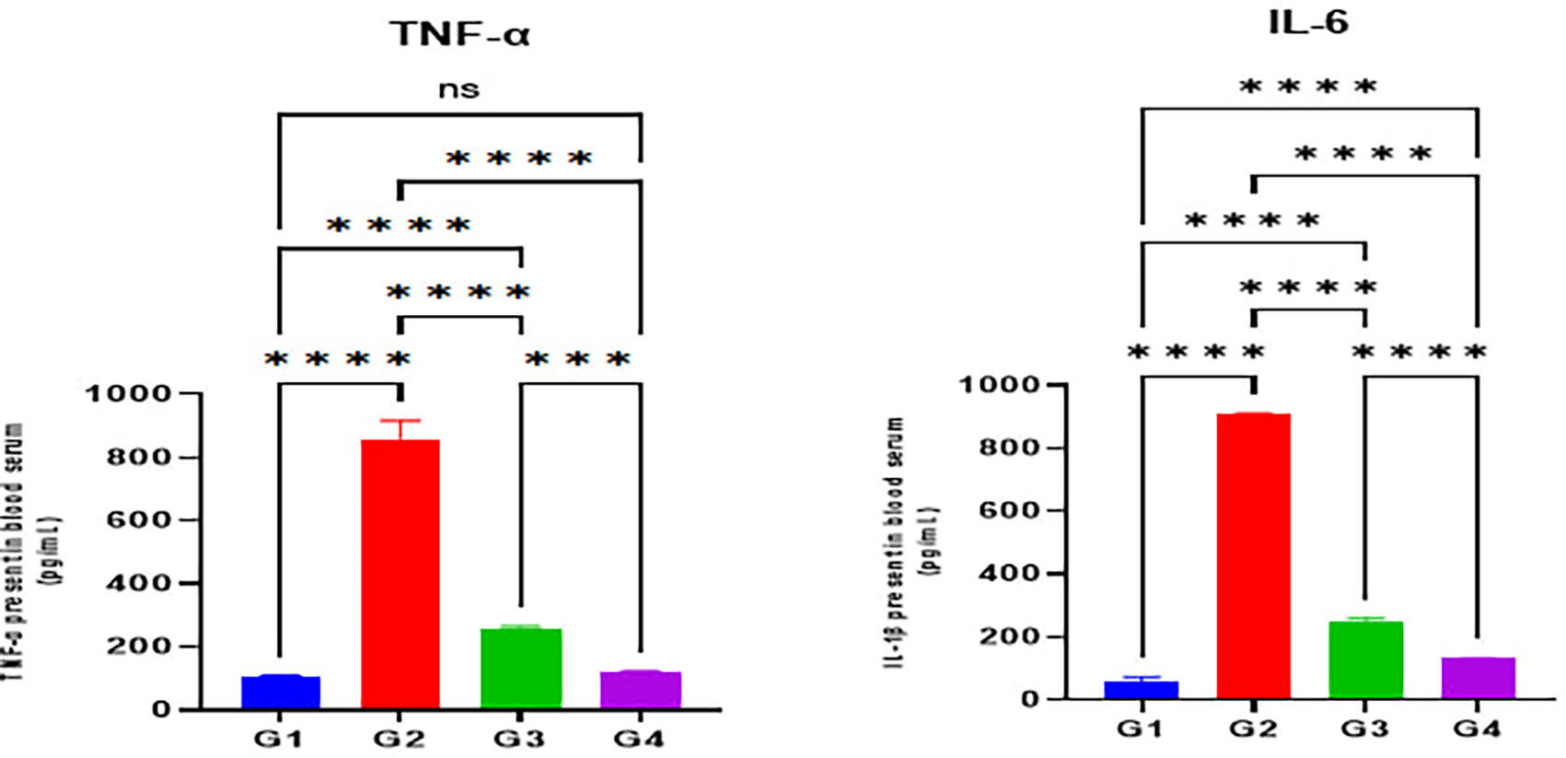
Effects of studied drugs on serum levels of inflammatory mediators (TNF-α and IL-1β) estimated at day 15 of the experiment for the control (G1), indomethacin induction (G2), omeprazole treatment (G3), and cranberry treatment (G4) groups. Data were presented as Mean±SD; **** = significant differences (p < 0.05), n = 5 animals/group.

Furthermore, serum levels of the inflammatory cytokine indicators TNF-α and IL-1β were substantially elevated in the indomethacin group (G2) when compared with the normal control group (G1) (p < 0.05). The omeprazole (G3) and cranberry (G4) treatment groups, however, presented a substantial reduction in serum levels of inflammatory markers TNF-α and IL-1β when compared with the indomethacin induction (G2) group (p < 0.05). On the other hand, it was demonstrated that the group treated with cranberry extract (G4) had substantially diminished serum levels of inflammatory mediators, including TNF-α and IL-1β, than the group treated with omeprazole (G3) (p < 0.05) as illustrated in Figure 3.

**Figure 4.**
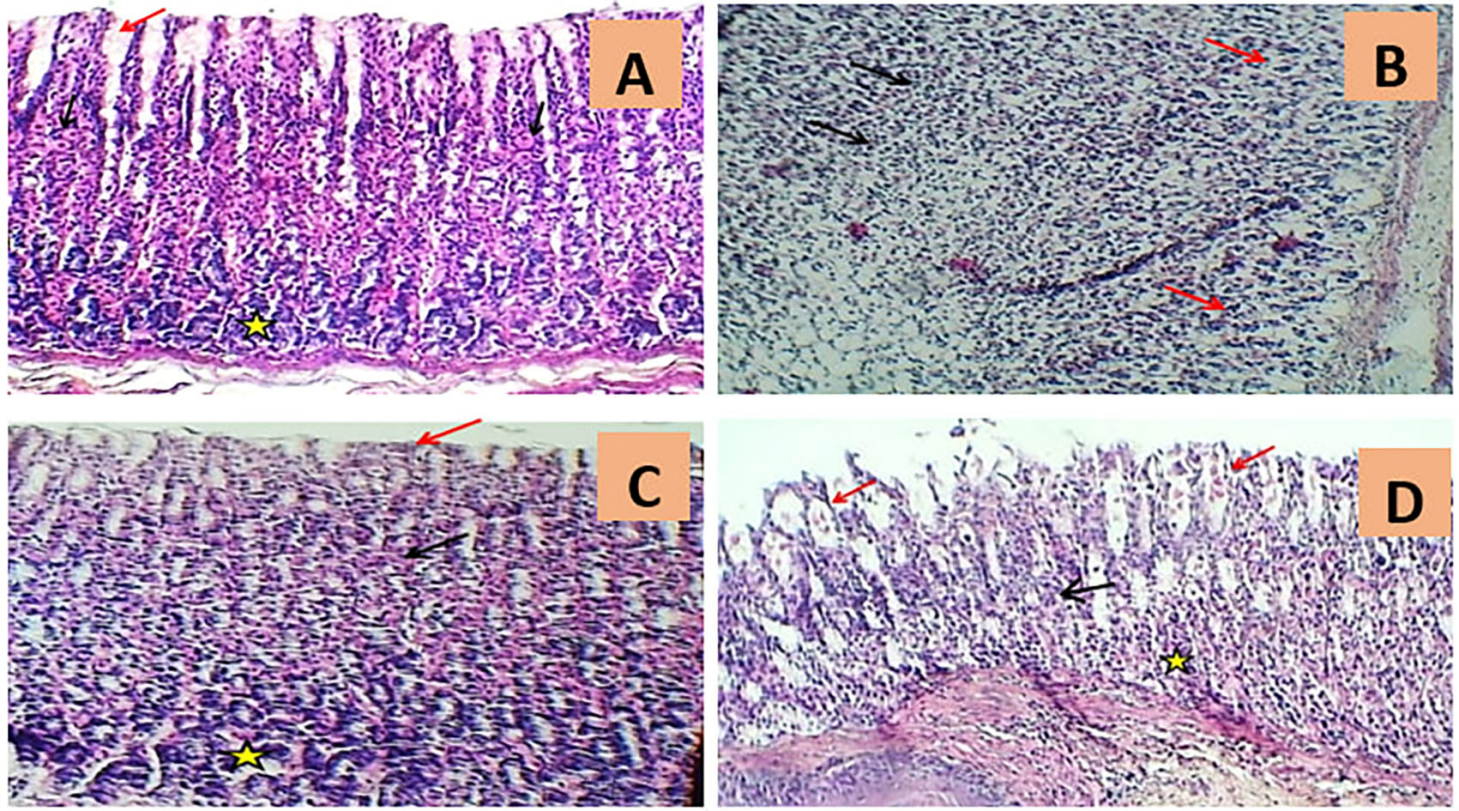
Exemplary photomicrographs of histopathological scores from different experimental groups of rats. A. The rat stomach histology of normal controls (G1) demonstrated the typical appearance of gastric pits (red arrow), parietal cells (black arrows), and chief cells (asterisk) (H&E 100X). B. The rat histological stomach segment of the indomethacin induction group (G2) exhibited severe massive necrotic gastritis characterized by marked mucosal surface necrosis and leukocyte infiltration (black arrows) and extensive degeneration of gastric glands (red arrows) (H&E 100x). C. The rat histological stomach segment treated with omeprazole (G3) displayed regular gastric pits (red arrow), parietal cells (black arrow), and chief cells of gastric glands (asterisk) (H&E 100X). D. The rat histological skin slice from the cranberry treatment group (G4) indicated intact gastric pits (black arrow), mild luminal sloughing of parietal cells (red arrows), and minimal cellular swelling of chief cells (asterisk) (H&E 100X).

In addition, the histological outcomes of the present investigation demonstrated that gastric specimens from normal rats had normal appearance of the stomach mucosa, submucosa, and muscularis, as clarified in Figure 4A and Table 1.

**Table 1.** Effects of studied drugs on the total histopathological scores of the stomach tissue in the control group (G1), indomethacin induction group (G2), Omeprazole treatment group (G3), and Cranberry treatment group (G4) on day 15 of the study.

Groups	Score in median (interquartile ranges)
**G1**	0(0-0.5)
**G2**	12(11.5-12) ^*^
**G3**	0(0-1) ^**^
**G4**	3(2.5-3.5)
**p-value **	0.000676

* Significant difference (p<0.05) versus control group (G1).

** Significant difference (p<0.05) versus indomethacin induction group (G2).

In contrast, the rat stomach tittue section of the indomethacin group (G2) enjoyed substantial histopathological abnormalities characterised by extensive mucosal surface necrosis, profound congestion with mononuclear leukocyte infiltration, and marked degeneration of digestive glands as compared to the control group (G1), as shown in Figure 4B and Table 1.

The omeprazole treatment group (G3), on the other hand, displayed a major reductionS in indomethacin-induced histopathological irregularities as evidenced by mild congestion, mild edema, minor gastric bleeding, moderate inflammatory cell infiltration, and slight necrotic changes, as seen in Figure 4C and Table 1.

The rat gastric section of the cranberry extract group (G4) indicated dramatically diminished histopathological modifications, including mild sloughing of parietal cells, minor cellular swelling of chief cells, little gastric haemorrhage with minimal leukocyte infiltration, congestion, and oedema. Yet, the cranberry extract group’s stomach mucosa seemed to be comparable to that of the ontrol group, exhibiting normal cytoarchitecture as illustrated in Figure 4D and Table 1.

## Discussion

It has been demonstrated beyond a reasonable doubt that oxidative stress and inflammation play a significant part in the etiology of indomethacin-induced injury to the stomach. ^86, 87^ In the current study, the level of antioxidant enzymes (SOD and GPX) was found to be considerably lower in the group that was subjected to ulcer induction (G2) compared to the group that served as the control (G1) and the other treatment groups (G3, G4). Under typical circumstances, the oxidant and antioxidant defense mechanisms of the organism are in equilibrium with one another for the group that serves as the control. ^88^ Diminished activities of SOD and GPx indicate impaired antioxidant pathways, which are frequently seen in many inflammatory diseases. ^89– 91^ These enzymes are necessary for neutralizing deleterious reactive oxygen species (ROS), and their absence can lead to further oxidative stress and inflammation. ^92– 95^

The injection of an induction dosage of indomethacin results in the production of ROS and produces oxidative stress. ^96^ According to research, indomethacin can bind to a region in the mitochondrial electron transport chain that is close to the complex and ubiquinone. This results in the uncoupling of the oxidative phosphorylation process and the formation of reactive oxygen species. ^97^ Consequently, reactive oxygen species (ROS) are responsible for the inactivation of mitochondrial aconitase, which leads to the generation of free iron, which in turn generates more mitochondrial •OH. ^98^ There is a correlation between oxidative stress and mitochondrial malfunction, the creation of mitochondrial permeability transition pores, and the generation of mitochondrial oxidative stress (MOS). ^99– 102^

Regarding the omeprazole treatment group (G3), there was a significant increase in the level of antioxidant enzymes (SOD and GPX) as compared to the ulcer induction group (G2). These findings are consistent with the findings of another research investigation, which demonstrated that omeprazole possesses antioxidant activity in addition to its antisecretory characteristics. Omeprazole was found to be a powerful scavenger of hypochlorous acid (HOCl) even at a drug concentration, and it also showed significant inhibition of iron- and copper-driven oxidant damage at pH 5.3 and 3.5, respectively, according to research that was carried out to investigate the in-vitro antioxidant effects of omeprazole at specific pH levels. ^103^ Another study discovered that OMP, because of its antioxidant activity, is characterized by the overexpression of superoxide dismutase in gastric mucosal cells. ^104^

The cranberry treatment group (G4) showed enhanced levels of antioxidant enzymes, SOD and GPX; as compared to the Indomethacin ulcer induction group (G2). Cranberry extract contains several bioactive compounds, such as anthocyanin and flavonoids, which are well known for their antioxidant activity. ^105^ Anthocyanins were found to induce the expression of several antioxidant enzymes, often mediated by Nrf2-dependent pathways responsible for inducing cytoprotective responses. In an earlier investigation to test the anti-oxidant function of Syzygium cumin (L.) Skeels on indomethacin-induced acute stomach ulceration, results indicated that the anthocyanin content of this plant dramatically upregulates SOD and GPx levels as compared to untreated rats. ^106^ Another study conducted to explore this finding found that the mRNA and protein levels of Nrf2 in anthocyanins treatment groups were near to the control group and higher than the NSAIDs- ulcer induction group. This is related to the ability of these phytochemicals to induce Nrf2 expression or inhibit its proteasomal degradation by modifying the Nrf2–Keap1 complex. ^107^

Several other flavonoids with antioxidant ability were reported in cranberries, such as rutin, apigenin, and quercetin. ^108^ Rutin is among the flavonoids that exert antioxidant and free radical scavenging activities. ^109, 110^ In a study investigating the antioxidant activity in rats with indomethacin-induced ulcers, rutin could significantly lessen the oxidative stress biomarkers deteriorated by indomethacin treatment. ^111^ Rutin has been proven in several laboratory investigations to boost glutathione levels and superoxide dismutase activity by capturing superoxide anions and scavenging free radicals, while also modulating TNF-α and IL-6 concentrations. ^112– 114^

Regarding quercetin, the most potent antioxidant flavonoid, studies demonstrated that it has a beneficial effect in attenuating indomethacin-induced gastric ulcers in rats by increasing the antioxidants enzymes activity (Catalase, SOD, and GPX). ^91, 94^ The mechanisms behind the antioxidant activity are scavenging oxygen radicals, protecting against lipid peroxidation, and chelating metal ions. ^115– 119^

In addition to oxidative stress, inflammation is also a crucial factor in the pathophysiology of gastropathy that is generated by nonsteroidal anti-inflammatory drugs. ^120– 122^ Based on the findings of the present study, it was observed that the level of inflammatory markers, specifically TNF-α and IL-1β, was considerably greater in the group that was subjected to ulcer induction (G2) compared to the group that served as the control (G1) and the other treatment groups (G3, G4). There is a direct connection between the formation of reactive oxygen species (ROS) and the oxidative stress that leads to a rise in the expression of TNF-α and IL-6 genes, which in turn leads to an increase in their levels through the nuclear factor kappa (NF-κB) dependent pathway. ^39, 123, 124^ More specifically, TNF-α acts to facilitate immune system responses and cellular proliferation. It encourages the translocation-related process of NF-κB, which aids communicating signals during inflammation. ^20, 125– 128^

Regarding the Omeprazole treatment group, there was a significant decrease in TNF-α and IL-6 levels compared to the ulcer induction group (G2). This indicates that omeprazole has an anti-inflammatory effect independent of suppressing gastric secretion. The suggested mechanism behind these effects is postulated to be related to the down-regulation of nuclear factor kappa (NF-κB) with subsequent suppression of pro-inflammatory cytokines, as reported by other researchers. ^129, 130^

Presently available therapeutic options for ulceration of the stomach feature a significant relapse probability. Natural remedies for ulceration management and therapy are not rare, since communities have historically used plant components. ^131^ A large fraction of the vegetable variety stays untapped for medicinal purposes. Antiulcer capabilities in botanical products exhibit potential, and many animal models are employed to assess their efficacy. ^132^ Competent experiments are required to evaluate the ulcer-preventing properties of botanicals and medicines. Such models are useful for understanding the pathological causes of wounds, as well as the antioxidative capabilities of critical medications or compounds with antiulcer effects. ^133^ There are several models for assessing anti-ulcer medications, rendering it difficult to pick an acceptable model.

The group that received cranberry treatment (G4) demonstrated a noteworthy reduction in the levels of TNF-α and IL-6 when compared to the levels that were observed in the group that was subjected to Indomethacin ulcer induction (G2). There is a connection between this discovery and the anti-inflammatory properties of cranberry extracts, which are primarily comprised of anthocyanins and proanthocyanins. ^39, 134^ The principal mechanisms by which anthocyanin compounds diminish inflammation involve hampering NF-κB, a transcriptional element that is vulnerable in terms of inflammatory and oxidative processes. ^135^ NF-κB is a substance produced by cells and located in the innermost part of the cell. It is inert due to its strong-affinity suppressor, IκB, which retains it in the cytoplasm and prevents its liberation. ^136^ Once triggering events, like as oxidative damage, occurs, a substantial signaling chain is initiated. The chain of reactions promotes IKK-a and IKK-b, two kinases that metabolize IκB. ^129^ Phosphorylation of IκB causes its disassociation, allowing NF-κB to relocate to the nucleus and attach to κB activation regions. This drives gene expression of chemotactic cytokines involving TNF-α and IL-1β. ^137^ Additionally, the phosphorylation of Mitogen-activated protein kinase (MAPKs) enzymes was suppressed by anthocyanin extract, and as a result, activation is necessary to mitigate the inflammatory response. ^138^ MAPKs, which include ERKs, c-JNKs, and p38, are a family of enzymes that react to various stimuli, one of which is inflammation. These enzymes, in turn, govern a wide variety of cellular responses, such as cell differentiation, mitosis, and apoptosis. ^139^ It is necessary to phosphorylate MAPKs for them to become active, as their base form is inactive from a catalytic perspective. ^140^

Furthermore, it has been proposed that the anti-inflammatory action that is brought about by cranberry extracts is due to the flavonoid component of the cranberry, which prevents neutrophils from initiating the infiltration process. ^141^ Several factors can lead to neutrophil infiltration, including the reduction in mucosal blood flow after IND administration. ^142^ According to the findings of a study, the flavonoid product known as rutin helped to boost the activity of cNOS, which in turn led to an increase in the levels of nitric oxide in the mucosal tissues of the stomach. Nitric oxide that is produced from cNOS can increase mucosal blood flow and tissue perfusion, which ultimately results in a significant reduction in neutrophil infiltration. ^143^

According to the results obtained, the highest ulcer index score was reported for the Indomethacin ulcer induction group (G2), which was significantly higher than the control, Omeprazole, and cranberry treatment groups. This is due to gastrotoxicity of a high dose of indomethacin inducing an inflammatory response and oxidative stress damaging the gastric mucosa coupled with decreasing the mucus and bicarbonate layer as a result of NSAIDs use which makes gastric tissue more liable to acidic damage of the gastric secretions. ^13^ On the other hand, the Omeprazole treatment group (G3) showed less ulcer score than the indomethacin ulcer induction group (G2) since omeprazole is a PPI that inhibits Na+/K+ ATPase enzyme leading to suppression of acid secretion. ^14^ Furthermore, from the previous results, omeprazole displayed antioxidant and anti-inflammatory actions that collectively contributed to their anti-ulcer effects. ^15^ The cranberry extracts treatment group showed an ulcer score that is significantly lower than the Indomethacin ulcer induction group (G2) and Omeprazole treatment group (G3) because of their potent antioxidant and anti-inflammatory effects owing to their flavonoid content. ^36^ The limitations of the current study could be summarized by the small number of animals used and the lack of estimation of the exact molecular mechanism through which cranberry extract exerts its gastro-protective effect. Estimation of Malondialdehyde (MDA) tissue levels, being the product of lipid peroxidation and the expression level of nuclear factor erythroid 2-related factor 2 (Nrf2) mRNA is usually used to predict the pathways of enhancing antioxidant activity while the nuclear factor kappa (NF-κB) mRNA to estimate the anti-inflammatory activity of the extract. Moreover, it is advised that future studies on female rats be conducted to identify gender-related differences. Furthermore, there is an urgent need for future research to explore the composition of phytomolecules present in cranberry extract using various separation techniques such as HPTLC-MS, NMR, LC-MS, etc.

## Conclusions

The current study’s findings revealed that cranberry extract proved its efficacy as a potential treatment for gastric ulcers caused by nonsteroidal anti-inflammatory drugs (NSAIDs) due to its anti-inflammatory and antioxidant properties, as confirmed by biochemical and histological analysis.

## Ethical approval

The research project has been approved by the Institutional Review Board (IRB) of the University of Baghdad’s College of Medicine. The Declaration of Helsinki’s requirements and guidelines were scrupulously adhered to in the course of conducting the current investigation. The ethical authority at University of Baghdad’s College of Medicine confirmed the required documentation and client data with approval number (UoB.Med.03-29) on December 31, 2023.

## Author contributions

**Zaid Mahmood Abdul Majeed** conducted the investigation, wrote and refined the first draft of the document, participated in its design and provided financing and other forms of assistance, donated supplies, equipment, and lab animals, and finished the final copy of the research article.

**Mohammed Qasim Yahya Malallah A. Al-atrakji** created the theoretical framework for the researched project, specified the parameters of the exploratory analysis, and outlined the main goals through an in-depth assessment of the findings, supplemented with insightful criticism and supervision.

## Data Availability

Figshare: The Potential Effects of Cranberry Extract on Indomethacin-induced Gastric Ulcers in Rats https://doi.org/10.6084/m9.figshare.28236107.v4 ^144^ This project contains following underlying dataset:
1. dr.zaid results.xlsx 2. histopathology of stomach.png Data are available under the terms of the Creative Commons Attribution 4.0 International license (CC-BY 4.0). Figshare repository: ARRIVE checklist for ‘Potential Effects of Cranberry Extract on Indomethacin-induced Gastric Ulcers in Rats’. https://doi.org/10.6084/m9.figshare.28236107.v4 ^144^ Data are available under the terms of the Creative Commons Attribution 4.0 International license (CC-BY 4.0).
