# Supplementary material for: The potential effects of cranberry extract on indomethacin-induced gastric ulcer in rats
Source: F1000Res. 2025 Jun 10;14:257. Originally published 2025 Mar 4. [Version 2] doi: 10.12688/f1000research.158944.2 (PMC12134734; doi:10.12688/f1000research.158944.2)
Supplement: Peer review history

# Reviewer report: Reviewer response for version 2

**DOI:** 10.5256/f1000research.183327.r390699

**Published:** 2025-06-25

**Authors:** Vishal Dubey

**Affiliations:** 1 Sundyota Numandis Probioceuticals Pvt. Ltd, Ahmedabad, Gujarat, India

The manuscript entitled “ **The potential effects of cranberry extract on indomethacin-induced gastric ulcer in rats**” aims to evaluate the effect of cranberry extract in preventing the severity of gastric mucosal ulcers resulting from indomethacin-induced gastric toxicity. In the revised manuscript, the authors have significantly improved overall readability and addressed most major queries. However, the following observations remain:

1. **Inconsistency in study timeline:** The manuscript mentions that the study was conducted between January and February 2024 in one section, and between January and June 2024 in another.
2. **Method of gastric tissue collection:** In the Methods section, describe how the gastric tissue was collected. If gastric tissue was stored for certain duration, then provide details of storage condition as well.
3. **Cytokine inconsistency (IL-6 vs IL-1ß):** Both IL-6 and IL-1ß are mentioned throughout the manuscript. Confirm which cytokine was actually studied and ensure consistency across the entire text.
4. **Histological scoring:** If only one histological slide was examined per group, clarify how the median and IQR of histological scores were calculated.
5. **Unexplained symbol in Figure 2:** The asterisk symbol (“**”) used in Figure 2 is not defined in the figure legend.
6. **“ns” in Figure 3:** The term “ns” appears in Figure 3 but is not defined in the figure legend.
7. **Inconsistent results reporting:** The Results section states that the levels of SOD and GPx in the cranberry and omeprazole groups are comparable (p > 0.05). However, Figure 2 suggests that the cranberry group shows significantly higher SOD and GPx levels than the omeprazole group.
8. **Labeling error in Figure 3:** The title of Figure 3 is “IL-6,” while the axis label refers to “IL-1ß.” Confirm whether IL-6 or IL-1ß.
9. **Contradiction in histological scores:** The Results section states that the histological score of the cranberry group is equal to that of the control group. However, Table 1 shows that the omeprazole group has a score equal to the control group.

Is the work clearly and accurately presented and does it cite the current literature?

Yes

If applicable, is the statistical analysis and its interpretation appropriate?

Yes

Are all the source data underlying the results available to ensure full reproducibility?

Partly

Is the study design appropriate and is the work technically sound?

Yes

Are the conclusions drawn adequately supported by the results?

Yes

Are sufficient details of methods and analysis provided to allow replication by others?

Partly

Reviewer Expertise:

Herbal and Dietary Supplements; Pharmacology.

I confirm that I have read this submission and believe that I have an appropriate level of expertise to confirm that it is of an acceptable scientific standard, however I have significant reservations, as outlined above.

# Reviewer report: Reviewer response for version 1

**DOI:** 10.5256/f1000research.174603.r385778

**Published:** 2025-06-03

**Authors:** Vijayakumar A. R

**Affiliations:** 1 Bharath Institute of Higher Education and Research, Chennai, Tamil Nadu, India

DEAR AUTHOR,

 The following point is to be clarified

 1. What is the rationale behind the use only in male animals?

 2. What is the normal lab temperature condition in Iraq (because 232°F was mentioned in the text)?

 3. Is there no significant effect on the control and cranberry-treated group in the TNF-alpha study (figure 3)?

 4. In the discussion, it was mentioned that the extract significantly reduced inflammatory markers, such as TNF-alpha; however, the result in Figure 3a showed no significant change in TNF-alpha.

Is the work clearly and accurately presented and does it cite the current literature?

Yes

If applicable, is the statistical analysis and its interpretation appropriate?

Yes

Are all the source data underlying the results available to ensure full reproducibility?

Yes

Is the study design appropriate and is the work technically sound?

Yes

Are the conclusions drawn adequately supported by the results?

Partly

Are sufficient details of methods and analysis provided to allow replication by others?

Yes

Reviewer Expertise:

Pharmacology and Toxicology

I confirm that I have read this submission and believe that I have an appropriate level of expertise to confirm that it is of an acceptable scientific standard, however I have significant reservations, as outlined above.

# Reviewer report: Reviewer response for version 1

**DOI:** 10.5256/f1000research.174603.r385782

**Published:** 2025-05-29

**Authors:** Vishal Dubey

**Affiliations:** 1 Sundyota Numandis Probioceuticals Pvt. Ltd, Ahmedabad, Gujarat, India

The manuscript entitled *“The potential effects of cranberry extract on indomethacin-induced gastric ulcer in rats”* aimed to evaluate the effect of cranberry extract in preventing the severity of gastric mucosal ulcers due to indomethacin-induced gastric toxicity. The authors have utilized histopathological tests and antioxidant and anti-inflammatory biomarkers to identify the effect of cranberry extract compared to appropriate standard, disease, and normal control groups. The results of the study are promising that supports the antioxidant and anti-inflammatory property of cranberry, further supporting its putative application in preventing NSAIDs induced gastric toxicity. While, the study is described in sufficient quantity and the observations of the study support the study conclusions, extensive language and grammatical errors observed in the entire manuscript. Great amount of work is required for updating the language of the manuscript. But certain major observations were identified as described below:

**ABSTRACT**

1. In methods section, it is mentioned that 24 rats were included and randomized in four groups with six rats per group, while in methods it is described that 20 rats were included with five rats per group.
2. In methods section, it is mentioned that rats were euthanized on day 16, while in manuscript it is mentioned that euthanasia was performed on day 15.

**INTRODUCTION**

1. Many words were identified for abbreviations which are not again used in the entire manuscript. For example: PGE2, VEGF, PDGF, bFGF. In academic writing, abbreviations must be identified for only those words that are being used for at least three or more times. Kindly check the entire manuscript for such abbreviations.
2. The abbreviations for indomethacin and omeprazole has been identified in the introduction section, but still many instances are observed that full word “indomethacin” and “omeprazole” instead of IND and OMP. In academic writing, the identification of abbreviations must ensure that the abbreviations are used consistently throughout the manuscript instead of the word. Kindly check the entire manuscript for such inconsistency.
3. The statement “… particularly polyphenols, which are required for the antioxidant activity of the fruit” can be rephrased as now it is providing the impression that only polyphenols are the components present in the fruit that is responsible for the antioxidant activity, while there are other ingredients as well (like citric and malic acids, vitamin c, and triterpenoids) that plays important role in the antioxidant effect of the fruit.
4. The statement “The traditional usage of cranberry for treating …. Treatment of NSAID-induced gastric ulcers” is confusing. Is the statement trying to explain the historical use of cranberry in disease conditions like UTI or describing the aim of the study. Separate statements are required for both the instances to prevent confusion.
5. The statement “As far as our knowledge, no previous studies were carried.” Is incomplete. What are the literature or scientific gap that is getting highlighted by this statement?

**METHODS**

1. The title of this section “methods” is identified twice. So suggestion to change the heading to “Materials” instead of “methods”
2. In experimental animals sub-section, any reason for including only male rats? If the study is based on any previous study methodology, then the limitations of that study methodology is required to be mentioned in the limitation section of this study as well, including this point as well.
3. The statement “They fasted for 24 hours ….free access to water.” does not belongs to this section and needs to be placed in the experimental design section.
4. Kindly provide the ethics committee approval number or protocol number for future study identification.
5. In drugs and reagents sub-section, the individual dose of IND and OMP (60 and 20 mg/kg) is not required to be mentioned in this section, as it will definitely let confusion on the strength of the drugs used.
6. Kindly mention the %strength of IND and OMP used in this study. Strength of drug (as %Assay) is crucial information that helps better replication of the current study in future.
7. Typo-error, use “marketed by Acino” instead of “marked by Acino”.
8. Identify the abbreviation (Na-CMC) for sodium carboxymethylcellulose in this section, as it is appreared for the first time in this section rather than in the latter sections. In academic writing, abbreviations must be identified on the first identification of the word and then abbreviation used consistently.

**METHODS**

1. The title of this section is already identified previously. Kindly make appropriate changes.
2. In preparation of pharmaceutical solutions sub-section, describe the method by which the IND suspension was prepared. It is just mentioned that IND suspension of strength 24 mg/ml was prepared but method is not described.
3. In preparation of pharmaceutical solutions sub-section, for OMP and cranberry solutions, commercially available products were utilized. But as pharmaceutical preparations comprises of excipients as well, was the method of solution preparation adjusted to meet the dose of 20 mg/kg for OMP and 200 mg/kg for cranberry. Or just the pharmaceutical preparation was suspended in Na-CMC and the prepared suspension was supplemented. In such case, the dose might not reach to the limit identified for OMP and cranberry. It is advised that in case the commercially available products are used as investigational therapy, detailed method of preparation is mandatory, along with mentioning the type of excipients are present in the formulation (if the data can be obtained from the manufacturer). The process of investigational therapy preparation is critical for study future replicability.
4. In preparation of pharmaceutical solutions sub-section, the statement “To provide a dose of …..a volume of 0.5 ml is required” can be rephrased as “To provide a dose of 20 mg/kg/rat, a dose of 0.5 ml of prepared suspension was administered”. Authors must always try to minimize the use of repetitive information in the entire manuscript, especially in the methods and results section, in this case is the average weight of rats which is already described in previous section. Similar observation is evident in following paragraphs as well, so changes in entire manuscript is expected.
5. In experimental design sub-section, the site “Iraqi Laboratory for Cancer and Biomedical Genome Research” is identified as one of the study sites, while in the previous experimental animals sub-section, the site “Iraqi Centre for Cancer Research and Medical Genetics” was identified as site for animal housing. Are both the sites same or different? If different, then the role of the site “Iraqi Laboratory for Cancer and Biomedical Genome Research” is required to be mentioned.
6. In experimental design sub-section, the study is identified to last till June 30, 2024, while in previous experimental animals sub-section, it is mentioned that study finished in February 2024. Authors necessarily will have to conduct rigorous evaluation of entire manuscript to identify and rectify such major inconsistencies.
7. In experimental design sub-section, identify the group abbreviations as G1, G2, G3, and G4 in this section rather than in the results section. For example, it can be written as Group 1 (G1; n=5)
8. In tissue and blood sample collection sub-section, the day of euthanasia is missing. As per abstract, euthanasia is performed on day 16, while anaesthesia is done on day 15. When was euthanasia done? If euthanasia is done on day 15, then were the animals supplemented with their designated therapy and then euthanasia performed, or if done in day 16, then were the animals fasted overnight?
9.  In tissue and blood sample collection sub-section, method of euthanasia is missing.
10. In tissue and blood sample collection sub-section, method of stomach and gastric tissue collection is missing.
11. In academic writing, numbers from 0-9 are written as full name (zero, one, …), while any other value is written as numerical. In tissue and blood sample collection sub-section, the value “three thousand revolutions” can be written as “3000 revolutions”.
12. In tissue and blood sample collection sub-section, the statement “Every attempt was established …..pain they may have experienced” can be removed, as this is the responsibility of the animal EC. Animal EC ensures this principle and only then provides the study approval. The description of animal EC role and responsibility in this section is not recommended.
13. In assessment of inflammatory and anti-oxidative parameters sub-section, the inflammatory biomarker evaluated were TNF-a and IL-1b or IL-6. In many instances (like in the results section), the use of IL-1b is identified, while in certain instances (like title of Figure 3), IL-6 is identified. Kindly confirm, IL-1b or IL-6, and make changes in entire manuscript accordingly.
14. In assessment of inflammatory and anti-oxidative parameters sub-section, what were the analytical sensitivities of the ELISA kits for the evaluated biomarkers. This is essential for study replicability.
15. In assessment of inflammatory and anti-oxidative parameters sub-section, were the collected serum directly utilized for biomarker analysis or further serum processing was performed. Kindly mention the same in this section, and if processing was performed than describe the method in sufficient detail to ensure future study replication.
16. In haematoxylin and eosin staining sub-section, identify the abbreviation H&E here rather than in the following sections.
17. In academic writing, the SI units are always written in abbreviated form and not mentioned in full form. So in the haematoxylin and eosin staining sub-section, the grams, and millilitres, litters can be written as gm, ml, L, respectively.
18. In assessment of histopathological changes sub-section, the statement “An experienced pathologist examined the treatment group without any prior knowledge” can be phrased as “An experienced pathologist who was blinded to the therapy in9tervention, examined the H&E stained tissues”.
19. In statistical analysis sub-section, it is defined that median and IQR was calculated for histological scores. But in previous sub-section, it is mentioned that only one sample slide was selected and evaluated from each group. How is it possible that only one sample is evaluated and median and IQR is calculated?

**RESULTS**

1. In Figure 2, the symbol ** in SOD graph is not defined in the figure legend. Also, it is advised that five * can be replaced with single * in the graph to make the figure less cluttered.
2. As per Figure 2, cranberry therapy showed significantly higher SOD and GPx levels compared to OMP group, while in results section it is mentioned that no difference between is observed between OMP and cranberry groups.
3. Consider changes in figure 3 similar as figure 2 regarding use of * instead of ****. Also, identify “ns” in the figure legend.
4. In figure 3, it is mentioned IL-6 as title, while figure Y-axis is termed as IL-1b.
5. In the statement “In contrast, the rat stomach tittue section ….”, try use alternative term to the word “enjoyed” as the described statement belongs to rats in the IND-group. Also, rectify the spell error “tittue” which is “tissue”.
6. As per table 1, the OMP group showed histological scores similar to normal control group, while in the results section, the statement “Yet, the cranberry extract group’s stomach ….. as illustrated in Figure 4D and Table 1” denotes that the cranberry group showed histological scores similar to normal control group.

**DISCUSSION**

1. More emphasis must be given on the strength and limitations of the study.
2. The study did not evaluated the effect of OMP + Cranberry therapy (to identify synergism or additive effect), the optimal dose of cranberry for preventing IND-induced gastric toxicity was not identified (only one dose was evaluated), the effect of therapies on symptomatic parameters (food and water intake level) was not presented. All these points are potential limitations of the current study. Identifying potential limitations of study does not down-grade the observations of the current study, but provides potential pathway for future experimental studies. It helps researchers identify the potential limitations, that might remain undermined if not elaborated clearly in this section.

Is the work clearly and accurately presented and does it cite the current literature?

Yes

If applicable, is the statistical analysis and its interpretation appropriate?

Yes

Are all the source data underlying the results available to ensure full reproducibility?

Partly

Is the study design appropriate and is the work technically sound?

Yes

Are the conclusions drawn adequately supported by the results?

Yes

Are sufficient details of methods and analysis provided to allow replication by others?

Partly

Reviewer Expertise:

Herbal and Dietary Supplements; Pharmacology.

I confirm that I have read this submission and believe that I have an appropriate level of expertise to confirm that it is of an acceptable scientific standard, however I have significant reservations, as outlined above.

# Reviewer report: Reviewer response for version 1

**DOI:** 10.5256/f1000research.174603.r374136

**Published:** 2025-04-17

**Authors:** Suraj N Mali

**Affiliations:** 1 Dr. D.Y. Patil Deemed to Be University, Navi, Mumbai, Maharashtra, India

Authors herein elaborated more on 'The potential effects of cranberry extract on indomethacin-induced gastric ulcer in rats'. However, I would like to see the phytomolecules present in them. In any way, authors can provide the composition by HPTLC-MS, NMR, LC-MS,etc.

 Why only male rats have been chosen? In order to see more gender specific outcomes, they can include Female as well. However, authors can give a proper reference with justification.

 A discussion part lacks detailing on phytocompositions. Please go through articles and update the literature in the introduction section: *Biol Trace Elem Res* (2024). [Ref 2]; [Ref 1]

Is the work clearly and accurately presented and does it cite the current literature?

Yes

If applicable, is the statistical analysis and its interpretation appropriate?

Yes

Are all the source data underlying the results available to ensure full reproducibility?

Yes

Is the study design appropriate and is the work technically sound?

Yes

Are the conclusions drawn adequately supported by the results?

Yes

Are sufficient details of methods and analysis provided to allow replication by others?

Yes

Reviewer Expertise:

Anti-Ulcer agents

I confirm that I have read this submission and believe that I have an appropriate level of expertise to confirm that it is of an acceptable scientific standard.

## References

[1] : From lab to nature: Recent advancements in the journey of gastroprotective agents from medicinal chemistry to phytotherapy. *Eur J Med Chem* .2024;272: 10.1016/j.ejmech.2024.116436 116436 10.1016/j.ejmech.2024.116436 38704935

[2] : Biogenic Nanoparticles as Safer Alternatives for Gastric Ulcers: An Update on Green Synthesis Methods, Toxicity, and Their Efficacy in Controlling Inflammation. *Biol Trace Elem Res* .2024; 10.1007/s12011-024-04446-4 10.1007/s12011-024-04446-4 39570521
